# Supplementary material for: Omega-3 Polyunsaturated Fatty Acids (PUFAs) and Diabetic Peripheral Neuropathy: A Pre-Clinical Study Examining the Effect of Omega-3 PUFAs from Fish Oil, Krill Oil, Algae or Pharmaceutical-Derived Ethyl Esters Using Type 2 Diabetic Rats
Source: Biomedicines. 2025 Jun 30;13(7):1607. doi: 10.3390/biomedicines13071607 (PMC12292556; doi:10.3390/biomedicines13071607)
Supplement: Supplementary file 1 [file biomedicines-13-01607-s001.zip › biomedicines-3670238-supplementary.pdf]

Supplemental Table S1: Fatty Acid % Composition of Diets

Diet	16:0	18:0	18:1	18:2	20:5	22:6
Control	21.3 ± 3.0	6.1 ± 0.9	30.8 ± 3.2	34.3 ± 5.1	< 1	< 1
High Fat	16.0 ± 1.2	12.6 ± 1.3	41.0 ± 3.6	23.4 ± 2.8	< 1	< 1
Menhaden oil	20.5 ± 1.4	8.0 ± 0.7	20.9 ± 2.3	14.8 ± 2.0	11.7 ± 1.5	6.5 ± 1.0
Krill oil	21.5 ± 1.7	14.1 ± 1.5	14.1 ± 1.9	4.7 ± 0.9	24.8 ± 2.3	13.1 ± 1.6
Algal oil EPA	18.2 ± 1.6	1.8 ± 0.4	9.0 ± 1.8	6.8 ± 1.4	32.1 ± 2.9	< 1
Algal oil DHA	17.0 ± 1.7	5.5 ± 0.7	20.3 ± 2.4	14.7 ± 1.9	< 1	17.2 ± 2.1
Algal oil EPA + DHA	18.1 ± 1.3	6.1 ± 0.6	27.7 ± 2.6	15.2 ± 0.9	10.0 ± 1.0	13.6 ± 1.4
Ethyl Ester EPA	7.1 ± 0.9	5.3 ± 0.6	17.3 ± 1.9	10.2 ± 1.4	44.7 ± 2.9	< 1
Ethyl Ester DHA	5.9 ± 0.7	3.6 ± 0.3	13.1 ± 1.3	7.8 ± 1.0	2.7 ± 0.7	53.8 ± 3.8
Ethyl Ester EPA + DHA	6.7 ± 0.6	4.2 ± 0.5	14.3 ± 1.5	8.8 ± 1.1	34.9 ± 2.7	14.9 ± 1.8

Data are presented as the mean of three determinations ± S.E.M.

Supplemental Table S2: Early Intervention Treatment with Menhaden oil, Krill oil, or Algal oils enriched in EPA, DHA or EPA + DHA of Type 2 Diabetic Rats: Effect on Body Weight, Blood Glucose, and Serum Free Fatty Acids, Triglycerides, Cholesterol Levels

Determination	Control (8)	Diabetic (9)	Diabetic + MO (9)	Diabetic + KO (9)	Diabetic + EPA (9)	Diabetic + DHA (9)	Diabetic + EPA + DHA (8)
Start weight (g)	336 ± 13	333 ± 10	325 ± 11	331 ± 11	325 ± 14	329 ± 10	332 ± 11
Final weight (g)	504 ± 7	402 ± 21[a]	394 ± 18[a]	435 ± 42[a]	406 ± 8[a]	395 ± 8[a]	433 ± 17[a]
Blood glucose (mg/dl)	146 ± 14	447 ± 29[a]	438 ± 29[a]	477 ± 46[a]	495 ± 20[a]	465 ± 16[a]	472 ± 31[a]
Free fatty acids (mmol/l)	0.05 ± 0.01	0.24 ± 0.03[a]	0.25 ± 0.05[a]	0.21 ± 0.03[a]	0.14 ± 0.03	0.12 ± 0.04	0.15 ± 0.03
Triglycerides (mg/dl)	41.9 ± 2.2	45.6 ± 0.4	44.4 ± 1.1	45.1 ± 0.9	44.2 ± 0.9	26.3 ± 2.9[a,b]	39.3 ± 3.5
Cholesterol (mg/ml)	1.9 ± 0.2	4.1 ± 0.7[a]	8.4 ± 2.2[a]	8.7 ± 2.8[a]	6.4 ± 1.2[a]	2.2 ± 0.3	2.9 ± 0.9

Data are presented as mean ± SEM. a $P < 0.05$ compared to control rats; b $P < 0.05$ compared to diabetic rats. Parentheses indicate the number of experimental animals.

Supplemental Table S3: Late Intervention Treatment with Menhaden oil, Krill oil, or Algal oils enriched in EPA, DHA or EPA + DHA of Type 2 Diabetic Rats: Effect on Body Weight, Blood Glucose, and Serum Free Fatty Acids, Triglycerides, Cholesterol Levels

Determination	Control (9)	Diabetic (9)	Diabetic + MO (9)	Diabetic + KO (9)	Diabetic + EPA (9)	Diabetic + DHA (9)	Diabetic + EPA + DHA (9)
Start weight (g)	331 ± 10	332 ± 9	325 ± 14	310 ± 7	317 ± 9	321 ± 9	318 ± 11
Final weight (g)	535 ± 11	404 ± 11[a]	370 ± 37[a]	418 ± 13[a]	407 ± 11[a]	416 ± 11[a]	425 ± 16[a]
Blood glucose (mg/dl)	157 ± 12	503 ± 23[a]	546 ± 18[a]	531 ± 9[a]	546 ± 15[a]	478 ± 13	478 ± 21[a]
Free fatty acids (mmol/l)	0.06 ± 0.01	0.25 ± 0.04[a]	0.21 ± 0.03[a]	0.22 ± 0.03[a]	0.24 ± 0.04[a]	0.09 ± 0.01[b]	0.14 ± 0.02
Triglycerides (mg/dl)	36.7 ± 3.7	52.7 ± 2.0[a]	52.6 ± 1.5[a]	50.2 ± 1.6[a]	51.8 ± 1.7[a]	29.4 ± 4.5[b]	39.7 ± 2.7[b]
Cholesterol (mg/ml)	1.8 ± 0.2	10.5 ± 3.1[a]	8.9 ± 2.6[a]	14.2 ± 3.2[a]	12.9 ± 3.1[a]	2.9 ± 0.5[b]	2.1 ± 0.3[b]

Data are presented as mean ± SEM. a $P < 0.05$ compared to control rats; b $P < 0.05$ compared to diabetic rats. Parentheses indicate the number of experimental animals.

Supplemental Table S4: Early Intervention Treatment with Menhaden oil, or Ethyl Esters of EPA, DHA or EPA + DHA of Type 2 Diabetic Rats: Effect on Body Weight, Blood Glucose, and Serum Free Fatty Acids, Triglycerides, Cholesterol Levels

Determination	Control (9)	Diabetic (9)	Diabetic + MO (9)	Diabetic + EPA (9)	Diabetic + DHA (9)	Diabetic + EPA + DHA (8)
Start weight (g)	331 ± 19	329 ± 21	326 ± 21	327 ± 22	335 ± 19	328 ± 20
Final weight (g)	492 ± 15	445 ± 18[a]	453 ± 24[a]	415 ± 10[a]	454 ± 18[a]	454 ± 18[a]
Blood glucose (mg/dl)	143 ± 9	489 ± 53[a]	445 ± 53[a]	492 ± 14[a]	449 ± 35[a]	487 ± 24[a]
Free fatty acids (mmol/l)	0.05 ± 0.01	0.24 ± 0.03[a]	0.25 ± 0.05[a]	0.21 ± 0.03	0.20 ± 0.03	0.19 ± 0.03
Triglycerides (mg/dl)	41.9 ± 2.2	45.6 ± 0.4	44.4 ± 1.1	45.7 ± 0.8	45.3 ± 1.2	44.1 ± 0.7
Cholesterol (mg/ml)	1.9 ± 0.2	4.1 ± 0.7[a]	8.4 ± 2.2[a]	5.2 ± 0.7[a]	3.6 ± 0.8	2.5 ± 0.3

Data are presented as mean ± SEM. a $P < 0.05$ compared to control rats; b $P < 0.05$ compared to diabetic rats. Parentheses indicate the number of experimental animals.

Supplemental Table S5: Late Intervention Treatment with Menhaden oil, or Ethyl Esters of EPA, DHA or EPA + DHA of Type 2 Diabetic Rats: Effect on Body Weight, Blood Glucose, and Serum Free Fatty Acids, Triglycerides, Cholesterol Levels

Determination	Control (9)	Diabetic (9)	Diabetic + MO (9)	Diabetic + EPA (8)	Diabetic + DHA (9)	Diabetic + EPA + DHA (9)
Start weight (g)	302 ± 12	303 ± 12	303 ± 13	302 ± 14	303 ± 13	302 ± 15
Final weight (g)	511 ± 13	435 ± 25	391 ± 18	405 ± 21	396 ± 19	439 ± 27
Blood glucose (mg/dl)	148 ± 6	460 ± 41[a]	541 ± 15[a]	455 ± 35[a]	531 ± 24[a]	415 ± 40[a]
Free fatty acids (mmol/l)	0.06 ± 0.01	0.25 ± 0.04[a]	0.21 ± 0.03[a]	0.22 ± 0.05	0.22 ± 0.04[a]	0.15 ± 0.02
Triglycerides (mg/dl)	36.7 ± 3.8	52.7 ± 2.0[a]	52.6 ± 1.5[a]	50.8 ± 3.0[a]	52.2 ± 2.0[a]	44.9 ± 0.8
Cholesterol (mg/ml)	1.8 ± 0.2	10.5 ± 3.1[a]	8.9 ± 2.6[a]	6.3 ± 1.7[a]	7.6 ± 2.4[a]	3.3 ± 0.6

Data are presented as mean ± SEM. a $P < 0.05$ compared to control rats; b $P < 0.05$ compared to Diabetic rats. Parentheses indicate the number of experimental animals.

Supplemental Table S6: Fatty Acid % Composition of Serum and Unsaturation Index after 12 Weeks of Treatment

Diet	16:0	18:0	18:1	18:2	20:4	20:5	22:5	22:6	Unsat. Index
Control	23.2 ± 1.3	13.2 ± 0.5	8.2 ± 0.4	20.9 ± 1.8	17.9 ± 0.8	1.7 ± 0.3	1.7 ± 0.3	3.9 ± 0.2	1.6 ± 0.1
Diabetic (Diab)	21.8 ± 0.4	19.4 ± 0.5[a]	12.1 ± 0.6[a]	20.5 ± 0.6[a]	17.7 ± 0.9	0.1 ± 0.1[a]	0.6 ± 0.1[a]	3.1 ± 0.2	1.5 ± 0.1[a]
Diab + Menhaden oil	23.1 ± 0.3	16.8 ± 0.4[a,b]	8.9 ± 0.3[b]	17.5 ± 0.3[a]	11.9 ± 0.5[a,b]	6.8 ± 0.3[a,b]	1.3 ± 0.1	7.5 ± 0.3[a,b]	1.7 ± 0.1[b]
Diab + Krill oil	23.0 ± 0.2	15.8 ± 0.8[b]	10.4 ± 0.6	20.9 ± 0.5	5.9 ± 0.3[a,b]	8.8 ± 0.7[a,b]	0.9 ± 0.1	8.5 ± 0.6[a,b]	1.8 ± 0.1[b]
Diab + Algal oil EPA	22.5 ± 0.7	16.8 ± 1.1[a]	10.0 ± 0.9	19.3 ± 0.8[a]	15.6 ± 1.4	5.9 ± 0.5[a,b]	2.9 ± 0.3[a,b]	2.0 ± 0.4	1.7 ± 0.1[b]
Diab + Algal oil DHA	18.8 ± 0.4[a,b]	20.4 ± 0.5[a]	5.2 ± 0.3[b]	9.2 ± 0.4[a]	24.0 ± 0.8[a,b]	0.9 ± 0.3	1.1 ± 0.2	10.8 ± 0.2[a,b]	2.2 ± 0.1[a,b]
Diab + Algal oil EPA + DHA	22.8 ± 0.7	16.5 ± 0.6[a]	9.2 ± 0.5[b]	15.1 ± 0.3[a]	12.7 ± 0.4[a,b]	7.3 ± 0.7[a,b]	1.8 ± 0.5[b]	10.0 ± 0.5[a,b]	2.0 ± 0.1[a,b]
Diab + Ethyl Ester EPA	22.2 ± 0.6	18.2 ± 0.8[a]	11.4 ± 0.6[a]	19.0 ± 0.3	14.3 ± 0.8	4.3 ± 0.3[a,b]	2.4 ± 0.1[a,b]	3.5 ± 0.4	1.6 ± 0.1
Diab + Ethyl Ester DHA	23.3 ± 0.6	17.9 ± 1.0[a]	10.5 ± 1.0[a]	22.0 ± 0.9	10.9 ± 0.9[a,b]	1.2 ± 0.1	0.6 ± 0.1[a]	9.1 ± 0.4[a,b]	1.6 ± 0.1
Diab + Ethyl Ester EPA + DHA	23.0 ± 0.2	18.0 ± 0.6[a]	11.1 ± 0.8[a]	20.0 ± 0.7	13.4 ± 0.9[b]	4.0 ± 0.3[a,b]	1.7 ± 0.1[b]	5.5 ± 0.5[b]	1.7 ± 0.1[b]

Data are presented as the mean of three determinations ± S.E.M. a $P < 0.05$ vs. Control, b $P < 0.05$ vs. Diabetic. Unsat. Index = Unsaturation Index

Supplemental Table S7: Fatty Acid % Composition of Liver and Unsaturation Index after 12 Weeks of Treatment

Diet	16:0	18:0	18:1	18:2	20:4	20:5	22:5	22:6	Unsat. Index
Control	20.8 ± 0.2	16.6 ± 0.5	7.7 ± 0.3	18.5 ± 0.4	21.3 ± 0.5	0.6 ± 0.1	1.8 ± 0.2	6.6 ± 0.3	1.84 ± 0.01
Diabetic (Diab)	18.1 ± 0.6[a]	21.4 ± 0.5[a]	9.7 ± 0.5[a]	12.2 ± 0.7[a]	22.8 ± 0.7[a]	0.5 ± 0.1	1.0 ± 0.1	6.7 ± 0.2	1.78 ± 0.02
Diab + Menhaden oil	20.3 ± 0.3	20.7 ± 0.6[a]	7.2 ± 0.3[b]	12.4 ± 0.1[a]	13.9 ± 0.2[a,b]	5.5 ± 0.3[a,b]	2.0 ± 0.1	14.5 ± 0.5[a,b]	2.15 ± 0.04[a,b]
Diab + Krill oil	21.4 ± 0.4	18.8 ± 1.1	8.6 ± 0.5	16.1 ± 0.6	8.6 ± 1.1[a,b]	6.2 ± 0.1[a,b]	1.7 ± 0.1	15.8 ± 1.7[a,b]	2.11 ± 0.05[b]
Diab + Algal oil EPA	20.6 ± 0.7	18.9 ± 0.9	8.8 ± 0.4	14.9 ± 0.5	17.1 ± 1.4[b]	4.7 ± 0.2[a,b]	6.6 ± 0.8[a,b]	5.4 ± 0.9	2.00 ± 0.06
Diab + Algal oil DHA	14.3 ± 1.4[a,b]	21.6 ± 0.7[a]	7.6 ± 1.2	7.5 ± 1.5[a,b]	19.0 ± 0.6	0.6 ± 0.1	8.6 ± 1.7[a,b]	20.8 ± 1.6[a,b]	2.53 ± 0.21[a,b]
Diab + Algal oil EPA + DHA	21.1 ± 1.0	22.3 ± 0.5[a]	9.6 ± 0.9	12.1 ± 0.7[a]	15.1 ± 0.4[a,b]	6.4 ± 0.5[a,b]	3.5 ± 1.3[b]	15.4 ± 1.0[a,b]	2.15 ± 0.16[a,b]
Diab + Ethyl Ester EPA	18.9 ± 0.4	22.2 ± 0.8[a]	8.7 ± 0.4	13.4 ± 0.6[a]	20.4 ± 1.3	5.2 ± 0.3[a,b]	3.1 ± 0.7[a,b]	6.6 ± 1.0	2.00 ± 0.04
Diab + Ethyl Ester DHA	20.2 ± 0.2	21.5 ± 0.8[a]	8.1 ± 0.4	15.2 ± 0.2	16.3 ± 1.2[a,b]	0.8 ± 0.6	0.7 ± 0.2	13.2 ± 1.1[a,b]	1.96 ± 0.02
Diab + Ethyl Ester EPA + DHA	19.1 ± 0.3[a]	20.7 ± 1.0[a]	9.7 ± 0.5	13.8 ± 0.2[a,b]	16.5 ± 0.4[a,b]	4.7 ± 0.9[a,b]	2.4 ± 0.2	12.2 ± 0.3[a,b]	2.02 ± 0.01

Data are presented as the mean of 8-10 determinations ± S.E.M. a $P < 0.05$ vs. Control, b $P < 0.05$ vs. Diabetic. Unsat. Index = Unsaturation Index
